# Reduced USP33 expression in gastric cancer decreases inhibitory effects of Slit2‐Robo1 signalling on cell migration and EMT

**DOI:** 10.1111/cpr.12606

**Published:** 2019-03-21

**Authors:** Yiwen Xia, Linjun Wang, Zhipeng Xu, Ruirui Kong, Fei Wang, Kai Yin, Jianghao Xu, Bowen Li, Zhongyuan He, Lu Wang, Hao Xu, Diancai Zhang, Li Yang, Jane Y. Wu, Zekuan Xu

**Affiliations:** ^1^ Department of Gastric Surgery The First Affiliated Hospital of Nanjing Medical University Nanjing China; ^2^ State Key Laboratory of Brain and Cognitive Science, Institute of Biophysics Chinese Academy of Sciences Beijing China; ^3^ Department of General Surgery Affiliated Hospital of Jiangsu University Zhenjiang China; ^4^ Department of Neurology, Center for Genetic Medicine Northwestern University Feinberg School of Medicine Chicago Illinois; ^5^ Department of Neurology Center for Genetic Medicine Lurie Cancer Center Chicago Illinois; ^6^ Jiangsu Key Lab of Cancer Biomarkers, Prevention and Treatment Jiangsu Collaborative Innovation Center for Cancer Personalized Medicine School of Publich Health Nanjing Medical University Nanjing China

**Keywords:** EMT, gastric cancer, migration and invasion, Robo1, Slit2, USP33

## Abstract

**Objectives:**

Gastric cancer (GC) is one of the most common cancers in the world, causing a large number of deaths every year. The Slit‐Robo signalling pathway, initially discovered for its critical role in neuronal guidance, has recently been shown to modulate tumour invasion and metastasis in several human cancers. However, the role of Slit‐Robo signalling and the molecular mechanisms underlying its role in the pathogenesis of gastric cancer remains to be elucidated.

**Materials and methods:**

Slit2, Robo1 and USP33 expressions were analysed in datasets obtained from the Oncomine database and measured in human gastric cancer specimens. The function of Slit2‐Robo1‐USP33 signalling on gastric cancer cells migration and epithelial‐mesenchymal transition (EMT) was studied both in vitro and in vivo. The mechanism of the interaction between Robo1 and USP33 was explored by co‐IP and ubiquitination protein analysis.

**Results:**

The mRNA and protein levels of Slit2 and Robo1 are lower in GC tissues relative to those in adjacent healthy tissues. Importantly, Slit2 inhibits GC cell migration and suppresses EMT process in a Robo‐dependent manner. The inhibitory function of Slit2‐Robo1 is mediated by ubiquitin‐specific protease 33 (USP33) via deubiquitinating and stabilizing Robo1. USP33 expression is decreased in GC tissues, and reduced USP33 level is correlated with poor patient survival.

**Conclusions:**

Our study reveals the inhibitory function of Slit‐Robo signalling in GC and uncovers a role of USP33 in suppressing cancer cell migration and EMT by enhancing Slit2‐Robo1 signalling. USP33 represents a feasible choice as a prognostic biomarker for GC.

## 1 INTRODUCTION

Gastric cancer (GC) is the third leading cause of cancer‐related death and responsible for approximately 723 000 deaths worldwide every year.^1^ Nearly half of the cases occur in Eastern Asia and are mostly diagnosed at the advanced stage.^2^ As a consequence, the 5‐year survival rate for advanced GC patients remains at only 5%‐20%.^3^ Hence, it is critical to explore the molecular mechanisms of GC development for finding new treatment strategy of GC.

Slit glycoproteins (Slit1‐3), originally discovered as neuronal guidance cues, are secreted by midline glia^4^ that exert their function by binding to single‐pass transmembrane proteins Roundabout family (Robo1‐4).^5^, ^6^, ^7^ The Slit‐Robo signalling pathway plays important roles not only in neuronal guidance but also during cell migration of a wide range of cell types.^6^, ^7^, ^8^, ^9^, ^10^ Recent studies indicate that the inactivation of this pathway is associated with the progression of several cancer types,^11^, ^12^, ^13^ including pancreatic cancer,^14^ breast cancer,^15^ as well as lung tumours.^16^ However, the precise function of the Slit‐Robo pathway in the development of GC remains ill‐defined. A number of studies supported the notion that Slit‐Robo signalling plays an important role in anti‐tumour processes.^17^, ^18^ In contrast, two other reports suggested that Robo1 might promote tumorigenesis.^19^, ^20^

Ubiquitin‐specific protease 33 (USP33), a member of ubiquitin‐specific protease family, was initially identified as a substrate molecule which binds to VHL E3 ligase.^21^ Previous studies showed that USP33 is a Robo1‐interacting protein that is involved in Slit signalling in midline axons crossing.^22^ Furthermore, USP33 is required for Slit‐Robo signalling in inhibiting breast cancer cell migration.^15^ Together, these studies demonstrate that USP33 plays an important role in the Slit‐Robo pathway.

Recently, a study based on data from one patient cohort reported that USP33 expression was found to be reduced in GC and that reduced USP33 expression was associated with poor prognosis.^23^ However, the precise molecular mechanisms of how USP33 exerts the anti‐tumour function in GC remain to be elucidated.

Here, we set out to investigate the role of Slit‐Robo signalling and the precise molecular mechanisms of how USP33 affects the Slit‐Robo signalling in GC.

## 2 METHODS

### 2.1 Clinical samples and cell culture

Primary GC samples were obtained from 54 patients who underwent radical resection for GC at the First Affiliated Hospital of Nanjing Medical University, China, between May 2016 and February 2017. No patient accepted adjuvant treatment for gastric cancer before surgery. Pathology and histology features of every case were confirmed by the Department of Pathology. Prior written informed consent from the patients or their relatives and approval from the Ethics Committee of the First Affiliated Hospital of Nanjing Medical University were obtained.

The human GC cell lines (MGC‐803, BGC‐823, HGC‐27, SGC‐7901 and AGS), the normal human gastric epithelial cell line GES‐1 and HEK‐293 cell line were purchased from the Shanghai Institutes for Biological Sciences, Chinese Academy of Sciences. All cells were cultured in DMEM (Gibco, USA) containing 10% foetal bovine serum (WISENT, Canada) and antibiotics (1% penicillin/streptomycin; Gibco) and incubated in a humidified chamber at 37°C under 5% CO_2_.

### 2.2 Antibodies and reagents

Anti‐Robo1 (ab7279), anti‐USP33 (ab71716), anti‐E‐cadherin (ab1416), anti‐N‐cadherin (ab18203), anti‐Snail (ab53519), anti‐Slug (ab27568), anti‐vimentin (ab8978) and anti‐GAPDH (ab8245) were purchased from Abcam (USA). Anti‐Flag (F3165) and cycloheximide (CHX, C7698) were obtained from Sigma‐Aldrich (USA). MG132 (HY‐13259) and chloroquine (HY‐17589) were obtained from MedChemexpress (USA).

### 2.3 RNA interference, plasmids and lentivirus transfection

The small interference RNA targeting USP33 (#1:5′‐GGAGAAUAGAUGUUCAUAUTT‐3′; #2:5′‐GCUGCAUUCAUCAAGUCAUTT‐3′) and a control siRNA (5′‐TTCTCCGAACGTGTCA CGTTT‐3′) were purchased from Gene Pharma Biotech (Shanghai, China). The lentiviral vector containing USP33 siRNA hairpin sequence and the puromycin resistance sequence (LV‐shUSP33) was also constructed by Gene Pharma. Slit2 tagged with c‐myc plasmid was generated as previously described.^7^ SiRNA and plasmids were transfected using Lipofectamine 2000 (Invitrogen, USA) according to the manufacturer's instructions.

### 2.4 RNA extraction and qRT‐PCR analysis

Total RNA was extracted from cells or frozen tissues with TRIzol reagent (Invitrogen), and then cDNA was synthesized using PrimeScript RT Master Mix kit (RR036A; Takara). The PCRs were then performed using the 7500 Real‐Time PCR System (Applied Biosystems, USA) with the primers as follows: Slit2 forward, 5′‐ACCGCTTCCAGTGCAAAGTA‐3′, reverse, 5′‐CTGGGTGCATGTCCCGTTAT‐3′; Robo1 forward, 5′‐GCATCCTCTCTGCCCTTCTC‐3′, reverse, 5′‐CTGGCTCGTGGAAGCTGTA A‐3′; USP33 forward, 5′‐AAAATCCCTTGGTACTTGTCAGG‐3’, reverse, 5′‐TCGAAGAGTGGTAAGGTTCACA‐3′; and GAPDH forward, 5′‐AGAAGGCTCATTTG‐3′, reverse, 5′‐AGGGGCCATCCACAGTCT TC‐3′.

### 2.5 Wound‐healing assay

Cell migration was examined in a modified wound‐healing assay. HEK293 cells which generate the full‐length Slit2 protein tagged with 6xMyc tag were cultured in DMEM with 5% FBS. The medium from HEK293 cells was used as a mock control. 3 × 10^5^ cells were grown in 6‐well plates until approximately 90% confluent. Then we used sterile 200 μL pipet tips to make the scratch at the centre of the plates. The cells were washed with PBS and then incubated in medium with or without Slit2. After a period of time, images were taken under a microscope and the distance between both sides was measured.

### 2.6 Transwell assay

To examine cell invasion, 24‐well BioCoat Matrigel Invasion Chambers (BD, Biosciences, Franklin Lakes, USA) were used according to the manufacturer's protocol. 3 × 10^4^ cells were cultured with serum‐free medium in the upper chamber, whereas medium containing 10% FBS was used in the lower chamber. The cells were incubated for 24 hour. Gently wiping the cells on the upper surface of the filters, cells on the lower surface were stained in 0.1% crystal violet (Sigma) for 30 minute. The number of invasion cell was then counted under microscope. The migration assay was conducted by the same methods, while the filters were not pre‐coated with Matrigel.

### 2.7 Western blot and immunoprecipitation

Total protein lysates were prepared with a protein extraction kit (KGP9100, Key Gene). Proteins were separated on 10% gels by SDS‐PAGE and transferred to polyvinylidene difluoride (PVDF) membranes. After blocking in 5% non‐fat milk in TBST buffer, the membranes were incubated with specific primary antibodies at 4°C overnight and followed by secondary antibodies. The signals were visualized using the chemiluminescence HRP substrate (WBKL0100; Millipore) and a chemiluminescence detection system.

Cell lysates were used for immunoprecipitation using the Dynabeads Protein G Immunoprecipitation kit (Invitrogen) following the manufacturer's guidelines. Immunoprecipitated proteins were then detected by Western blot.

### 2.8 Immunohistochemical analysis

All specimens were fixed in 4% formalin and then embedded in paraffin. The 4 μm sections were incubated with primary antibodies at 4°C overnight. After washing with PBS, the sections were incubated with HRP‐polymer‐conjugated secondary antibody at room temperature for 1 hour. Next, sections were stained with DAB solution for 3 minutes and the nuclei were counterstained with haematoxylin. The results were evaluated by both the intensity of cell staining (graded as 0, no staining; 1, weak; 2, moderate; and 3, strong) and the percentage of positive tumour cells (graded as 0, <5%; 1, 5%‐25%; 2, 26%‐50%; 3, 51%‐75%; and 4, >75%). Intensity score and percentage score were calculated.

### 2.9 Immunofluorescence microscopy

Cells were cultured on collagen‐coated glass coverslips for 24 hour and then rinsed with PBS twice before fixation with 4% formaldehyde for 20 minute at 37°C. Subsequently, cells were rinsed with PBS for three times and permeabilized with 0.2% Triton X‐100 for 10 minute. The cells were incubated with PBS containing 1% BSA for 30 minute and then incubated with the primary antibody at 4°C overnight. Afterwards, cells were washed and incubated with fluorophore‐conjugated secondary antibodies (Cy3™‐Goat Anti‐Rabbit IgG or Cy2™‐Goat Anti‐Mouse IgG Jackson Immunoresearch) for 2 hour and then stained with DAPI for 5 minute. After the final wash, a fluorescent microscope was used (Nikon, Japan) to collect images.

### 2.10 Animal studies

BALB/c nude mice (4 weeks old, female) were purchased from the Department of Laboratory Animal Centre of Nanjing Medical University. Control MGC‐803 or BGC‐823 cells, cells stably expressing Slit2 with shControl or shUSP33 (1 × 10^6^ cells in 100μl PBS) were injected into the caudal vein of anesthetized nude mice (6 mice per group). Mice were monitored using an in vivo imaging systems (IVIS) (Caliper Life Sciences, USA). Six weeks following tumour injection, mice were euthanized with lung tissues collected for haematoxylin‐eosin staining and analyses.

### 2.11 Statistical analysis

All data were analysed using SPSS 20.0 software (SPSS Inc, Chicago, IL, USA). The results obtained from cell line experiments and animal assays were analysed using Student's *t* test (for two groups) or ANOVA (for more than two groups). Mann‐Whitney *U* test was used to analyse differences in immunohistochemical (IHC) scores. Chi‐square test was used to analyse association of the expression of Robo1 and USP33 with clinicopathologic features. The Kaplan‐Meier method was used the survival analyses. The optimal cut‐off values of USP33 expression were generated by X‐tile software. Data are presented as the mean ± SD. *P < *0.05 was considered significant.

## 3 RESULTS

### 3.1 Expression of Slit2 and Robo1 is down‐regulated in gastric cancer

**Figure 1:**
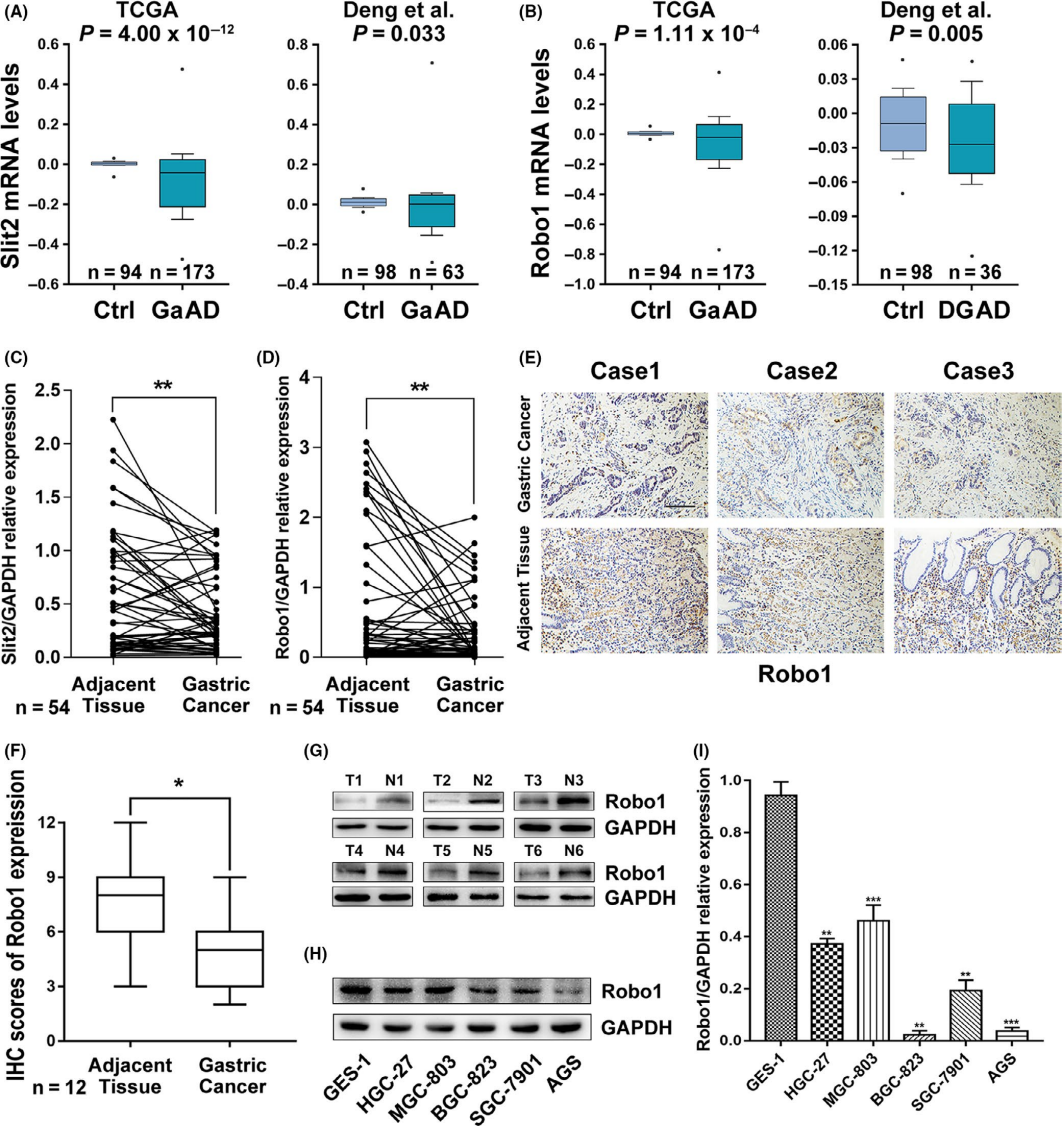
Expression of Slit2 and Robo1 is down‐regulated in gastric cancer. (A and B) Slit2 expression and Robo1 expression were analysed in TCGA and Deng's datasets from Oncomine (http://www.oncomine.org). Box and whisker plots: line represents the median value, boxes show 25th and 75th percentiles, whiskers mean 10th and 90th percentiles and the dots indicate maximum and minimum values. *P*‐values were calculated from Oncomine software using Student's *t* test. Ctrl, control gastric tissues; GaAD, gastric adenocarcinoma; DGAD, diffuse gastric adenocarcinoma. (C) Slit2 mRNA expression in 54 paired GC and adjacent tissues analysed by qRT‐PCR. (D) Robo1 mRNA expression in 54 paired GC and adjacent tissues analysed by qRT‐PCR. (E) Representative images of immunohistochemical (IHC) staining of Robo1 in 12 paired GC and adjacent tissues. Original magnification, 200×; scale bar: 100 µm. (F) Box plots showing the IHC scores for Robo1 protein expression, analysed by Mann‐Whitney *U* test. (G) Robo1 protein levels in 6 random paired GC and adjacent tissues determined by Western blotting. (H) Robo1 protein expression in 5 gastric cell lines and the normal human gastric epithelial cell line GES‐1 detected by Western blotting. (I) Robo1 mRNA expression in 5 gastric cell lines and the normal human gastric epithelial cell line GES‐1 detected by qRT‐PCR

To investigate the role of Slit‐Robo family in GC, we first measured Slit2, 3 and Robo1, 2, 3 expression in 54 paired cancer tissues and matched adjacent non‐cancer tissues from GC patients. Slit1 was excluded for its limited expression in nervous tissues, and Robo4 was excluded for lacking of Slit binding site. It was found that Slit2 and Robo1 showed the most significant decrease in GC (Figure 1A,B and Figure S1A). Meanwhile, Slit2 and Robo1 were also decreased in patients of stage III than patients of stage I and II (Figure S1B,C), suggesting its potential role in the development in GC. We then examined Slit2 and Robo1 expression in datasets from the Oncomine database (http://www.oncomine.org/).^24^ The mRNA levels of both Slit2 and Robo1 are lower in GC samples when compared with the control samples in the TCGA and Deng's datasets (Figure 1C,D).

We next examined Robo1 protein levels in 12 pairs of GC samples using immunohistochemistry. Robo1 expression was significantly lower in GC tissues compared with matched non‐cancer tissues. The representative images and the IHC scores are shown in Figure 1E,F. In agreement with above results, Western blot in six pairs of GC samples also indicated that Robo1 protein levels were lower in GC tissues (Figure 1G).

We also determined the Robo1 mRNA and protein levels in normal human gastric epithelial cell line (GES‐1) and five GC cell lines (HGC‐27, MGC‐803, BGC‐823, SGC‐7901 and AGS; Figure 1H,I). Both mRNA and protein levels of Robo1 in GC cell lines were found to be lower than those determined for GES‐1.

### 3.2 Slit2 inhibits GC cell migration in a Robo‐dependent manner and suppresses EMT

**Figure 2:**
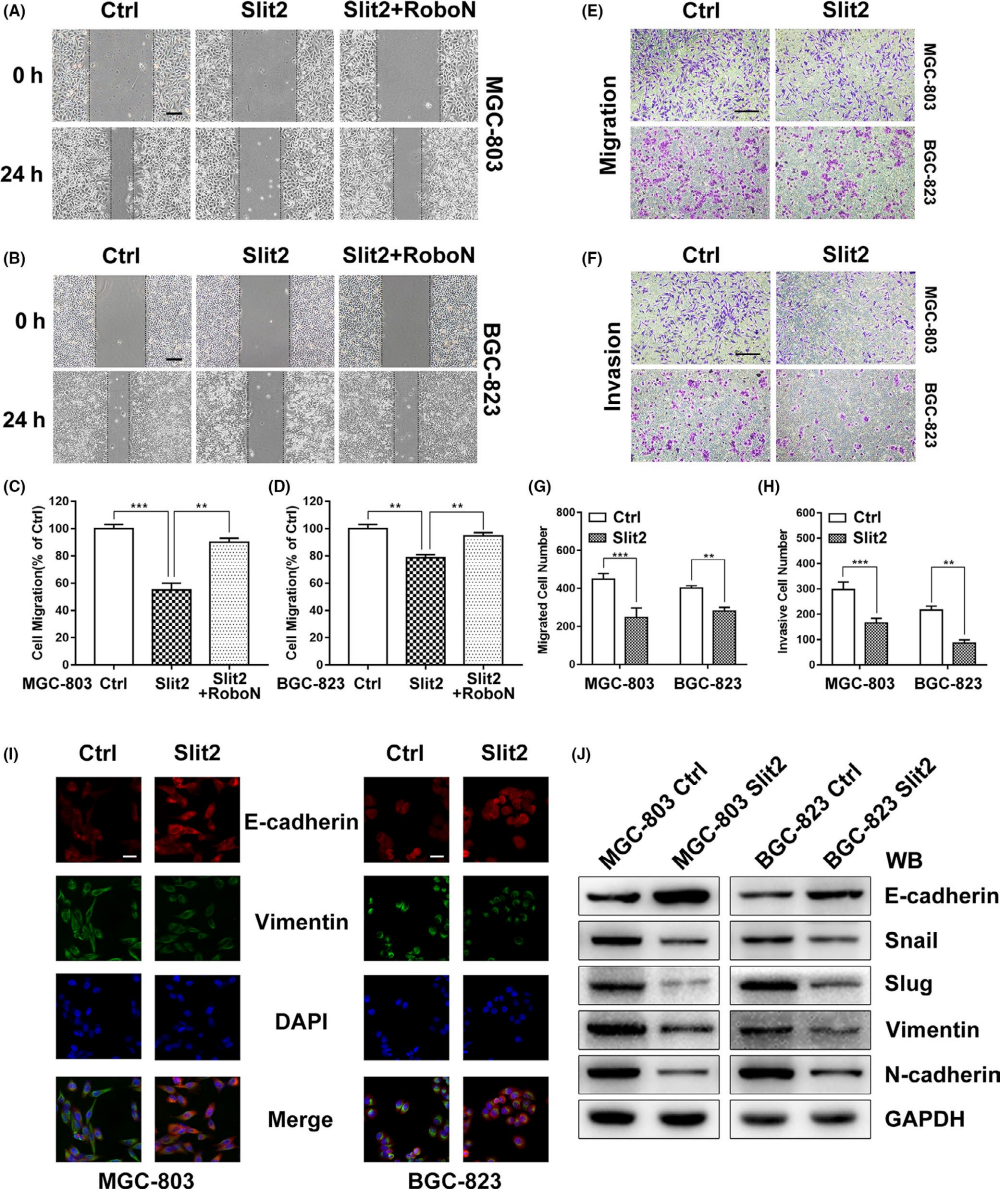
Slit2 inhibits migration of GC cells in a Robo‐dependent manner and suppresses EMT markers. A, Cell migration was examined in a wound‐healing assay using MGC‐803 cells in the medium containing the mock control (Ctrl), Slit2 and Slit2 with RoboN. Original magnification, 40×; scale bar: 100 µm. B, The migration of BGC‐823 cells tested by wound‐healing assays. C, Quantification of the distance of MGC‐803 cell migration. D, Quantification of the distance of BGC‐823 cell migration. E, Cell migration was examined in MGC‐803 and BGC‐823 cells transfected with Slit2 plasmid or control in a transwell assay. Original magnification, 100×; scale bar: 200 µm. F, Cell invasion was examined in MGC‐803 and BGC‐823 cells in the transwell assay. G, Cell migration was quantified. H, Cell invasion was quantified. I, Immunofluorescent microscopy was used to detect expression of E‐cadherin (red) and vimentin (green) in MGC‐803 and BGC‐823 transfected with Slit2 plasmid or control, DAPI (blue) was applied for nuclear staining. Original magnification, 400×; scale bar: 50 µm. J, The expression of epithelial cell marker (E‐cadherin), mesenchymal cell markers (N‐cadherin, Vimentin) and related transcription factors (Snail, Slug) was analysed by Western blotting. GAPDH was used as an internal control. All data are shown as mean ± SEM and analysed by Student's *t* test, *P < 0.05, **P < 0.01, ***P < 0.001

To investigate the role of Slit2‐Robo1 signalling in GC progression, we used two independent GC cell lines, MGC‐803 and BGC‐823 expressing Robo1 receptor for the following studies (Figure 1H). We performed a wound‐healing assay to examine the role of Slit2 in GC cell migration. Slit2 treatment suppressed MGC‐803 cell migration compared with the control media (Figure 2A,C). To evaluate the involvement of Robo1 in Slit2 signalling, RoboN, the soluble extracellular domain of Robo1 that blocks Slit‐Robo signalling,^6^, ^7^ was used in the wound‐healing assay together with Slit2. RoboN effectively blocked the inhibitory effect of Slit2 on MGC‐803 cell migration (Figure 2A,C). Consistent with data from MGC‐803 cells, BGC‐823 cell migration was also suppressed by Slit2 in a Robo‐dependent manner in the wound‐healing assay (Figure 2B,D). We next examined the effect of Slit2 using a transwell assay following transfection with Slit2 expressing plasmid or a control vector. Slit2 expression reduced cell migration and invasion of both MGC‐803 and BGC‐823 in the transwell assay (Figure 2E‐H).

It is well documented that epithelial‐mesenchymal transition (EMT) is a critical process in cell invasion and metastasis.^25^, ^26^ We first examined the epithelial marker E‐cadherin and the mesenchymal marker vimentin by Western blot and immunohistochemistry in GC samples. Compared with the normal tissues, E‐cadherin protein levels were lower in GC samples, while vimentin levels were found to be elevated (see Figure S1D‐H).

To examine the potential role of Slit2 signalling in EMT of gastric cancer, we examined the expression markers in MGC‐803 and BGC‐823 transfected with Slit2 plasmid or control by immunofluorescent microscopy. Slit2 overexpression increased expression of E‐cadherin and suppressed expression of vimentin (Figure 2I). In agreement with immunofluorescence analysis, Western blot showed that Slit2 overexpression increased the levels of epithelial cell marker (E‐cadherin) while reducing the expression of mesenchymal cell markers (N‐cadherin, vimentin), as well as related transcription factors (Snail, Slug; Figure 2J).

Together, these results clearly demonstrated that Slit2 inhibits GC cell migration and invasion in a Robo‐dependent manner and suggest that Slit2 signalling suppresses EMT in GC.

### 3.3 USP33 expression is down‐regulated in GC and correlates with Robo1 expression

Our previous studies suggested that USP33 regulates the expression of Robo1 and is therefore essential for the activation of the Slit‐Robo pathway.^15^, ^27^, ^28^ The observation that Robo1 expression is reduced in GC samples prompted us to examine whether USP33 affects the development of GC.

**Figure 3:**
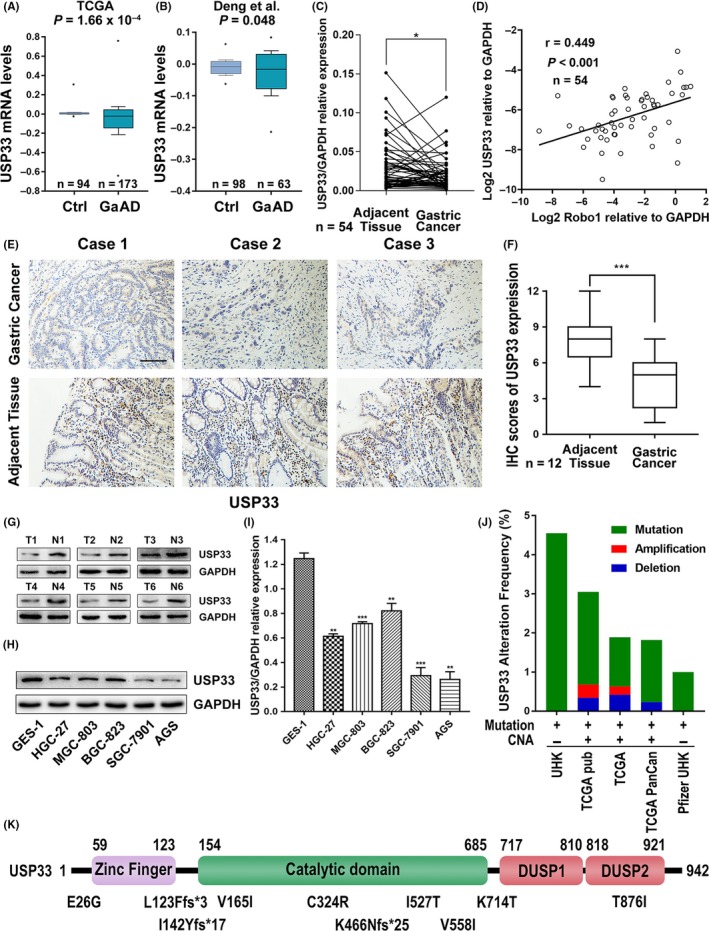
USP33 expression was down‐regulated in GC, and USP33 was correlated with Robo1 expression. (A and B) USP33 expression was analysed in TCGA and Deng's datasets from Oncomine. (C) USP33 mRNA expression in 54 paired GC and adjacent non‐cancer tissues analysed by qRT‐PCR. (D) Linear regression analysis was used to examine the correlation between Robo1 and USP33 mRNA expression levels in human GC tissues. *r* = 0.449, *P* < 0.001, n = 54. (E) Representative images of IHC staining of USP33 in 12 paired GC and adjacent tissues. Original magnification, 200×; scale bar: 100 µm. (F) Box plots showing the IHC scores for Robo1 protein expression, analysed by Mann‐Whitney *U* test. (G) USP33 protein levels in 6 paired GC tissue (T) and adjacent non‐cancer tissue samples (N) were determined by Western blotting. (H) USP33 protein expression in five gastric cell lines and the normal human gastric epithelial cell line GES‐1 detected by Western blotting. (I) USP33 mRNA expression in 5 gastric cell lines and the normal human gastric epithelial cell line GES‐1 detected by qRT‐PCR. (J) Copy number alterations (CNA) and frequency of USP33 gene mutations or deletion in different datasets from cBioPortal (http://www.cbioportal.org). (K) Distribution of USP33 mutations in gastric adenocarcinoma across protein domains

Analysis of USP33 expression in the public GC datasets showed that USP33 is commonly down‐regulated in GC samples (Figure 3A,B). We further examined the USP33 mRNA expression in our own cohort of paired GC samples. Unsurprisingly, USP33 mRNA expression was also found to be lower in GC tissues (Figure 3C). Moreover, linear regression analysis revealed that the relative expression levels of USP33 correlated well with Robo1 (Figure 3D). USP33 protein levels were also reduced in GC tissues by both immunohistochemistry and Western blot analysis (Figure 3E‐G). Consistently, both mRNA and protein levels of USP33 were lower in GC cell lines relative to those found in the control GES‐1 line (Figure 3H,I).

To assess the overall frequency of genetic alterations of USP33 in GC patients, we analysed large datasets from cBioPortal for Cancer Genomics (http://cbioportal.org).^29^ As shown in Figure 3J, mutations of the USP33 gene in GC patients were detected in five independent cohorts, ranging from 1% to 4.55%, while copy number alteration (CNA) was observed in three cohorts. Interestingly, ten USP33 mutations were identified in GC patient samples, with five mutations inside the catalytic domain of USP33 and two additional frameshift (fs) mutations upstream of the catalytic domain (Figure 3K).

### 3.4 USP33 interacts with Robo1 in GC cells

**Figure 4:**
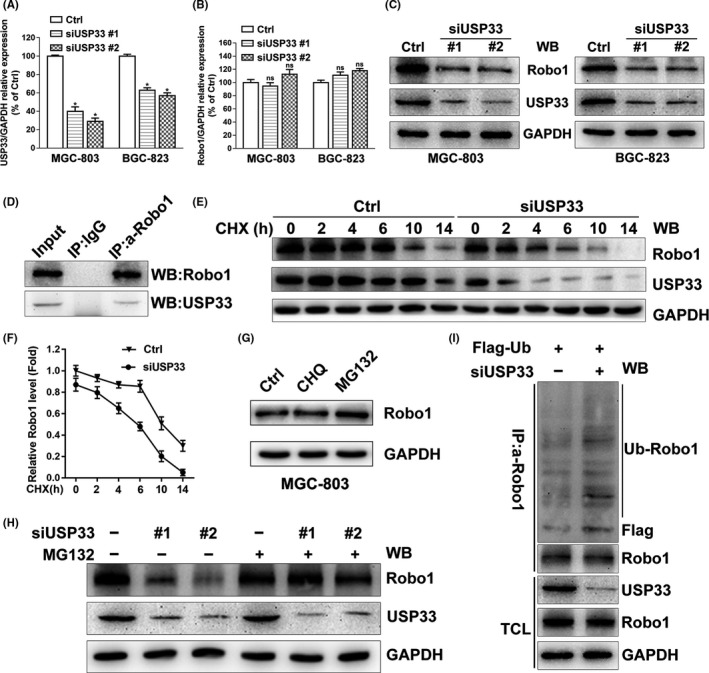
USP33 interacts with Robo1 and increases the stability of Robo1 by deubiquitinating Robo1 in GC cells. A, Relative USP33 mRNA levels in MGC‐803 and BGC‐823 transfected with control siRNA, siUSP33 #1 or siUSP33 #2 were examined by qRT‐PCR. B, Relative Robo1 mRNA levels in MGC‐803 and BGC‐823 cells transfected with control siRNA or siUSP33. C, Western blotting showed the protein levels of USP33 and Robo1 in MGC‐803 and BGC‐823 cells transfected with control siRNA or siUSP33. D, Interaction of the Robo1 and USP33 proteins in MGC‐803 cells. Co‐immunoprecipitation was performed using either control IgG or anti‐Robo1 antibody. Immunoprecipitated proteins were detected by Western blotting using anti‐Robo1 and anti‐USP33. E, MGC‐803 cells were transfected with control siRNA or siUSP33 and treated with cycloheximide (CHX, 50 μg/mL) for different periods of time. The Robo1 and USP33 protein levels were subjected to Western blotting analysis. F, Quantification of relative Robo1 protein levels. G, MGC‐803 cells were left untreated or treated with chloroquine (CHQ, 50 μmol/L, 10 h) or MG132 (10 μmol/L, 10 h), and Robo1 was detected by Western blotting. H, MGC‐803 cells transfected with control siRNA or siUSP33 were treated with MG132 (10 μmol/L) or untreated, 10 hours later, and Robo1 and USP33 were detected from the cell lysate. I, Robo1 ubiquitination was examined in MGC‐803 cells co‐transfected with Flag‐ubiquitin, control siRNA or siUSP33. Co‐immunoprecipitation was carried out with anti‐Robo1 after treated with MG132 (10 μmol/L, 10 h) and then examined by Western blotting. TCL: Total cell lysate. All Western blotting analyses in this figure 4 were using GAPDH as the internal control

To examine the relationship between USP33 and Robo1, we transfected MGC‐803 and BGC‐823 with two different siRNAs against USP33 (siUSP33 #1 and #2). Quantitative RT‐PCR analysis showed that the USP33 siRNAs reduced mRNA expression of USP33 (Figure 4A). Robo1 mRNA levels, however, were not affected by USP33 siRNAs (Figure 4B). In comparison, Western blot showed that USP33 knock‐down caused a decrease in both USP33 and Robo1 protein levels (Figure 4C), suggesting that USP33 regulates Robo1 protein expression through post‐translational modification.

We next examined whether USP33 interacts with Robo1 in GC cells by co‐immunoprecipitation assay. While the control antibody showed no precipitation, the anti‐Robo1 antibody specifically co‐immunoprecipitated USP33, indicating that USP33 interacts with Robo1 in MGC‐803 cells (Figure 4D). To test whether Slit2 affects Robo1‐USP33 interaction, co‐immunoprecipitation experiments were carried out using untreated MGC‐803 cells, cells treated with Slit2 containing media or cells transfected with Slit2 plasmid. Robo1 and USP33 were determined in immunoprecipitated proteins by Western blot. In comparison, neither Slit2 treatment nor expression failed to affect interaction between Robo1 and USP33 (see Figure S2A).

### 3.5 USP33 deubiquitinates and stabilizes Robo1

We next examined whether USP33 affects the stability of Robo1. MGC‐803 cells were transfected with either siUSP33 or control siRNA (Ctrl), and treated with cycloheximide (CHX, an inhibitor of protein synthesis). We then performed a time‐course experiment to measure the protein levels as a function of time. As shown in Figure 4E,F, Robo1 levels decreased considerably 2 hour post‐CHX treatment. At 14 hour post‐treatment, Robo1 protein was almost completely degraded in MGC‐803 cells transfected with siUSP33 compared with the control sample. These results showed that USP33 knock‐down shortens the half‐life of Robo1, indicating that USP33 stabilizes Robo1 by reducing the degradation of Robo1 protein.

The main proteolytic systems responsible for intracellular protein degradation are the ubiquitin‐proteasome system (UPS) and the lysosomal system.^30^ To examine the role of lysosomes vs the UPS system in Robo1 degradation, MGC‐803 cells were treated with chloroquine (CHQ, a lysosome inhibitor) or MG132 (a proteasome inhibitor). The Robo1 protein level increased after MG132 treatment, whereas chloroquine treatment failed to show visible effects (Figure 4G). These effects were also confirmed in additional 4 GC cell lines (see Figure S2B), suggesting that Robo1 is degraded mainly via the ubiquitin‐proteasome system in GC cells.

Furthermore, the decrease in the Robo1 protein level induced by siUSP33 was blocked by MG132 (Figure 4H). We then examined the levels of ubiquitinylated Robo1 after co‐transfecting Flag‐tagged ubiquitin (Flag‐Ub) together with either control siRNA (Ctrl) or siUSP33. Downregulation of USP33 increased the level of ubiquitinylated Robo1 in the presence of MG132 (Figure 4I).

Together, these data support the notion that USP33 stabilizes Robo1, preventing it from ubiquitin‐proteasome‐mediated degradation.

### 3.6 USP33 mediates Slit2 signalling in inhibiting GC cell migration and EMT process in vitro

**Figure 5:**
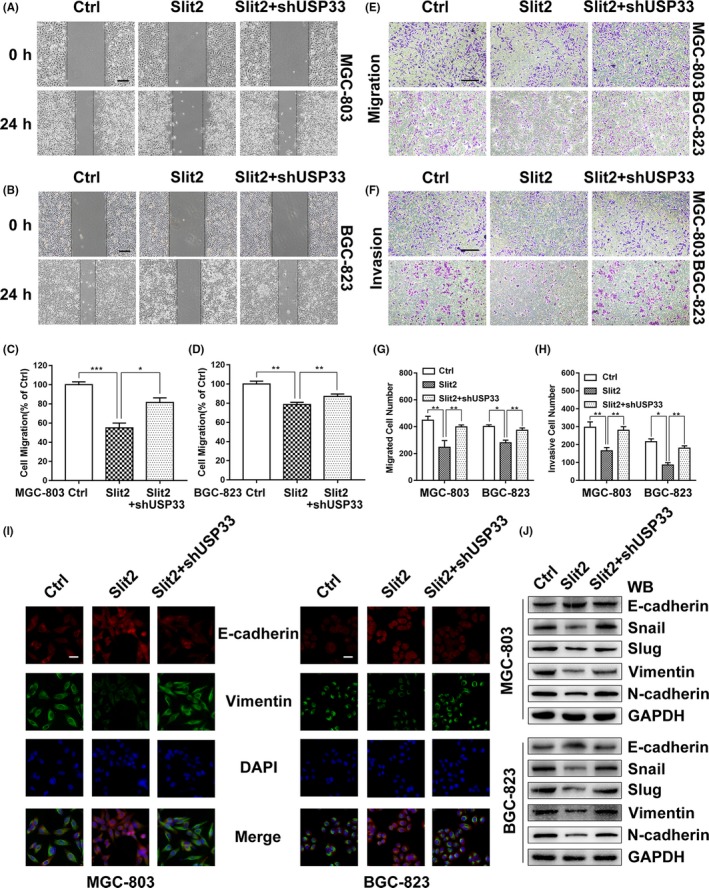
USP33 mediates Slit2 signalling in inhibiting GC cell migration and EMT in vitro*. *A, The migration of MGC‐803 cells expressing Slit2, Slit2 + shUSP33 or the control was examined in the wound‐healing assay. Original magnification, 40×; scale bar: 200 µm. B, The migration of BGC‐823 cells was examined in the wound‐healing assay. C, Quantification of the migration distance of MGC‐803 cells. D, Quantification of the migration distance of BGC‐823 cells. E, The migration of MGC‐803 and BGC‐823 cells was examined in the transwell assay. Original magnification, 100×; scale bar: 200 µm. F, Cell invasion by MGC‐803 and BGC‐823 cells was examined in the transwell assays. G, The number of migrated cells was counted. H, The number of invaded cells was counted. I, Immunofluorescent microscopy was used to detect expression of E‐cadherin (red) and vimentin (green) in MGC‐803 and BGC‐823 cells expressing Slit2, Slit2 + shUSP33 or the control, and DAPI (blue) was applied for nuclear staining. Original magnification, 400×; scale bar: 50 µm. J, The expression of epithelial cell marker (E‐cadherin), mesenchymal cell markers (N‐cadherin, Vimentin) and related transcription factors (Snail, Slug) was analysed by Western blotting. GAPDH was used as an internal control. All data are shown as mean ± SEM and analysed by Student's *t* test, *P < 0.05, **P < 0.01, ***P < 0.001

To test the involvement of USP33 in Slit2‐Robo1 signalling, we performed a wound‐healing assay. USP33 expression was reduced in GC cells by lentiviral vector containing a small hairpin sequence targeting USP33 (LV‐shUSP33). The efficiency of transfection was confirmed by qRT‐PCR and Western blot (see Figure S2C,D). As shown in Figure 5A‐D, Slit2 overexpression suppressed GC cell migration, however USP33 knock‐down blocked the inhibitory function of Slit2 on cell migration in both MGC‐803 and BGC‐823 cells. Similar to the results of wound‐healing assay, the transwell assay also showed that the inhibitory effects of Slit2 in GC cell migration and invasion were reversed upon USP33 knock‐down (Figure 5E‐H). It should be noted that USP33 knock‐down by itself did not affect cell migration or invasion (see Figure S2E‐H).

To investigate the role of USP33 in Slit2 signalling in EMT, we examined the EMT markers in MGC‐803 and BGC‐823 cells using immunofluorescent microscopy and Western blot. Slit2 overexpression increased the expression of the epithelial marker E‐cadherin and reduced the expression of the mesenchymal marker vimentin (Figure 5I). In addition, USP33 knock‐down by shUSP33 attenuated effects of Slit2 in increasing expression of E‐cadherin and decreasing Vimentin. Furthermore, our Western blot analysis showed that Slit2 overexpression increased E‐cadherin levels, but decreased expression of mesenchymal cell markers (N‐cadherin, Vimentin) and related transcription factors (Snail, Slug) (Figure 5J). Similarly, these Slit2‐induced effects were diminished upon USP33 knock‐down.

Together, these results clearly demonstrate that USP33 mediates Slit2 signalling in inhibiting GC cell migration and EMT in cultured cells.

### 3.7 USP33 mediates the inhibitory function of Slit2 signalling on metastasis in vivo

**Figure 6:**
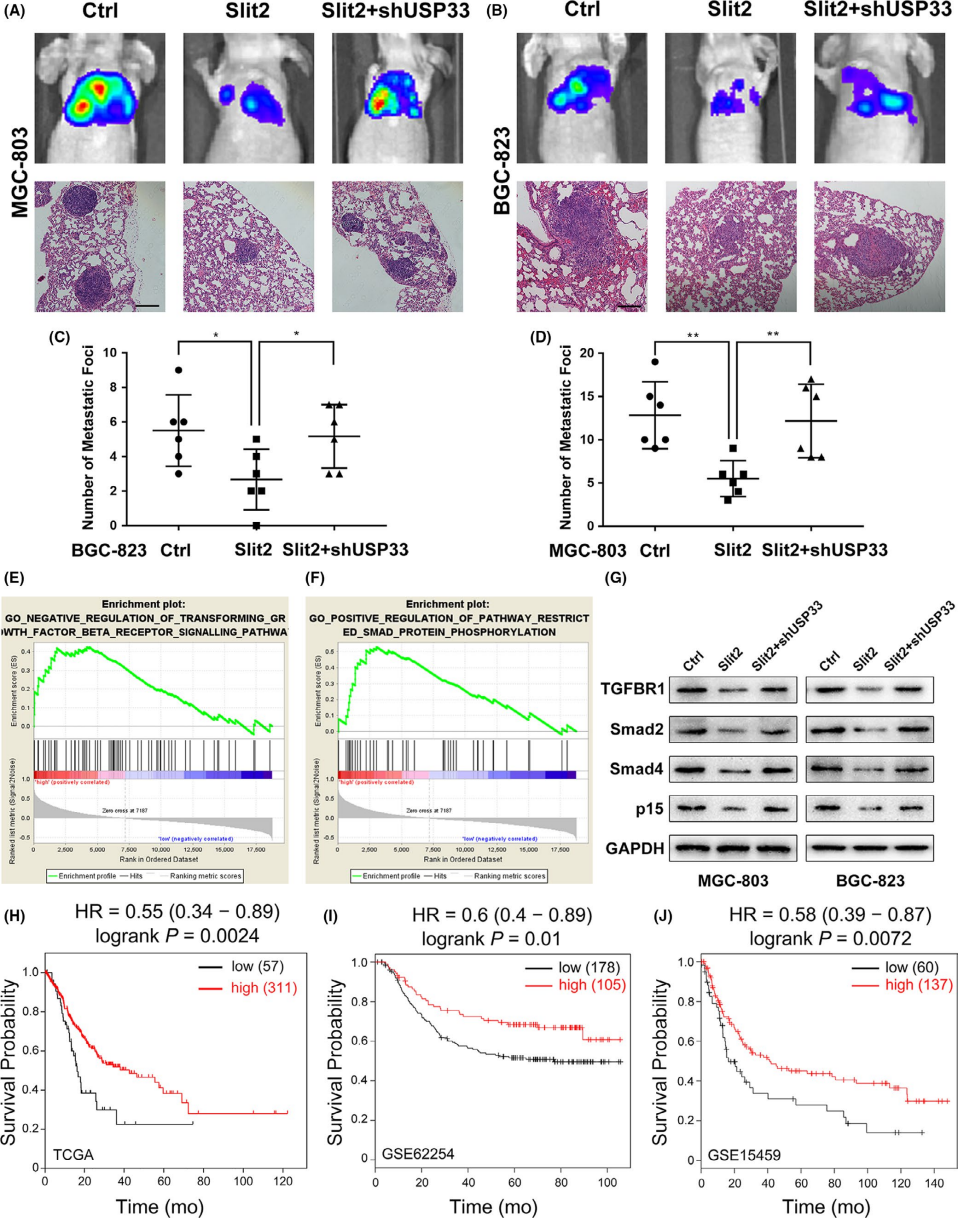
USP33 mediates the inhibitory function of Slit2 signalling in GC metastasis in vivo*, *and low USP33 expression predicts shorter patient survival. (A) MGC‐803 cells stably expressing Slit2, Sli2 + shUSP33 or the control (1 × 10^6^ cells in 100 μL PBS) were injected into the tail vein of 4‐week‐old female BALB/c nude mice (6 mice per group). Tumour progression and metastases were monitored using an IVIS Imaging system. Mice were euthanized 6 weeks after injection, and the lung tissues were harvested for haematoxylin‐eosin (HE) staining. Representative images of bioluminescent images of mice and HE staining of lung tissues were shown. Original magnification, 200×; scale bar: 100 µm. (B) Representative images of bioluminescent images of mice and HE staining of lung tissues injected with BGC‐823. Original magnification, 200×; scale bar: 100 µm. (C) Quantification of metastatic foci in mice injected with MGC‐803 cells. (D) Quantification of metastatic foci in mice injected with BGC‐823 cells. (E, F) A gene set enrichment analysis (GSEA) was performed to compare the Slit2 higher group (red) against Slit2 lower group (blue) with GC cohorts of the TCGA database. Higher Slit2 expression correlates with the negative regulation of transforming growth factor β receptor signalling pathway and positive regulation of pathway restricted Smad protein phosphorylation. (G) Expression of key proteins involved in TGFβ pathway by Western blotting. (H‐J) Patient survival analyses based on USP33 expression in three independent datasets, TCGA, GSE62254 and GSE15459. The overall survival analyses were displayed with hazard rate and log‐rank test *P*‐values

To investigate the role of Slit2 and USP33 in GC metastasis, we used an in vivo xenograft model. Control MGC‐803 or BGC‐823 cells, cells stably co‐expressing Slit2 with shControl or shUSP33 were injected into the caudal veins of athymic BALB/c nude mice (6 mice per group). Mice were monitored for 6 weeks using an IVIS Imaging system. Six weeks after tumour cell injection, mice were euthanized with the lung tissues harvested for histological examination. The numbers of lung metastatic foci were quantified (Figure 6C,D), and representative images are shown in Figure 6A,B. Overexpression of Slit2 significantly inhibited GC metastasis of both MGC‐803 and BGC‐823 cells. Knock‐down of USP33 in Slit2 expressing GC cells, however, diminished the inhibitory effects of Slit2 on tumour metastasis.

Together, these results strongly suggest that USP33 mediates Slit2 signalling in inhibiting GC metastasis in vivo.

### 3.8 USP33 is required for Slit2‐Robo1 signalling in inhibiting TGF‐β pathway

To further elucidate the potential pathway regulated by Slit2‐Robo1 signalling, a gene set enrichment analysis was performed using Slit2 expression as a phenotype label in GC cohorts from TCGA database. Higher Slit2 expression was significantly correlated with negative regulation of TGF‐β pathway and pathway that restricted Smad protein phosphorylation (*P* < 0.05; Figure 6E,F). Transforming growth factor‐β (TGF‐β) is widely upregulated in several human cancers^31^ and could promote invasion and metastasis by inducing EMT in cancer cells,^32^ while the phosphorylation of Smad proteins plays a key role in the TGF‐β pathway.^33^ These data suggested that Slit2 may inhibit migration and EMT via inhibiting TGF‐β pathway in GC.

We then measured the key proteins involved in TGF‐β pathway. Western blot showed that Slit2 reduced the expression of TGF‐β receptor I (TGFBR1), p15 (a downstream target of TGFβ), Smad2 and Smad4 in MGC‐803 and BGC‐823, while these effects were attenuated by USP33 knock‐down (Figure 6G).

These results indicated that USP33 is required for Slit2‐Robo1 signalling in inhibiting TGF‐β pathway.

### 3.9 USP33 expression is inversely correlated with tumour size, lymph node metastasis and neural invasion in GC, and low USP33 expression predicts poor survival

To explore the clinical significance of USP33, we examined the correlation between the USP33 expression and clinicopathological characteristics in our GC cohort. As shown in Table 1, USP33 expression was inversely correlated with tumour size, lymph node metastasis and neural invasion. From TCGA dataset, higher USP33 expression significantly correlates with longer overall survival (Figure 6H). Furthermore, KM‐plotter analysis of additional GC datasets^34^ also shows that high USP33 expression was associated with extended patient survival in two independent datasets, GSE62254 and GSE15459 (Figure 6I,J). Together, these results suggest that USP33 represents a suitable choice as a prognostic marker for GC.

**Table 1:** Correlation between USP33 mRNA expression and the clinicopathological characteristics in 54 paired GC patients

Characteristics	Number	USP33 expression		*P*‐value
		Low group	High group	
Age (y)				
<60	17	10	7	0.559
≥60	37	17	20	
Gender				
Male	38	19	19	0.999
Female	16	8	8	
Size (cm)				
<3	19	5	14	0.021*
≥3	35	22	13	
Histology grade				
Well‐*moderately*	37	21	16	0.241
Poorly signet	17	6	11	
T grade				
T1 + T2	24	9	15	0.170
T3 + T4	30	18	12	
Lymph node metastasis				
Absent (N0)	22	5	17	0.002*
Present (N1‐N3)	32	22	10	
Stage				
I/II	32	12	20	0.051
III/IV	22	15	7	
Blood vessel invasion				
Absent	35	15	20	0.254
Present	19	12	7	
Neural invasion				
Absent	27	7	20	0.001*
Present	27	20	7	

*Chi‐square *test was performed to analyse the correlations,

* *P* < 0.05.

## 4 DISCUSSION

Gastric cancer remains a common cause of tumour‐related death and a major health problem in the world, especiallyin Eastern Asia.^1^, ^2^ Although great efforts have been made, the mechanisms underlying the tumorigenesis and development of GC remain implicit, which partially accounts for the poor prognosis of GC patients. Epithelial‐mesenchymal transition (EMT) has emerged as a critical process of cell invasion and metastasis in most epithelial tumour, including GC,^25^, ^26^ and could serve as a potential target for cancer pharmacological intervention.^35^

**Figure 7:**
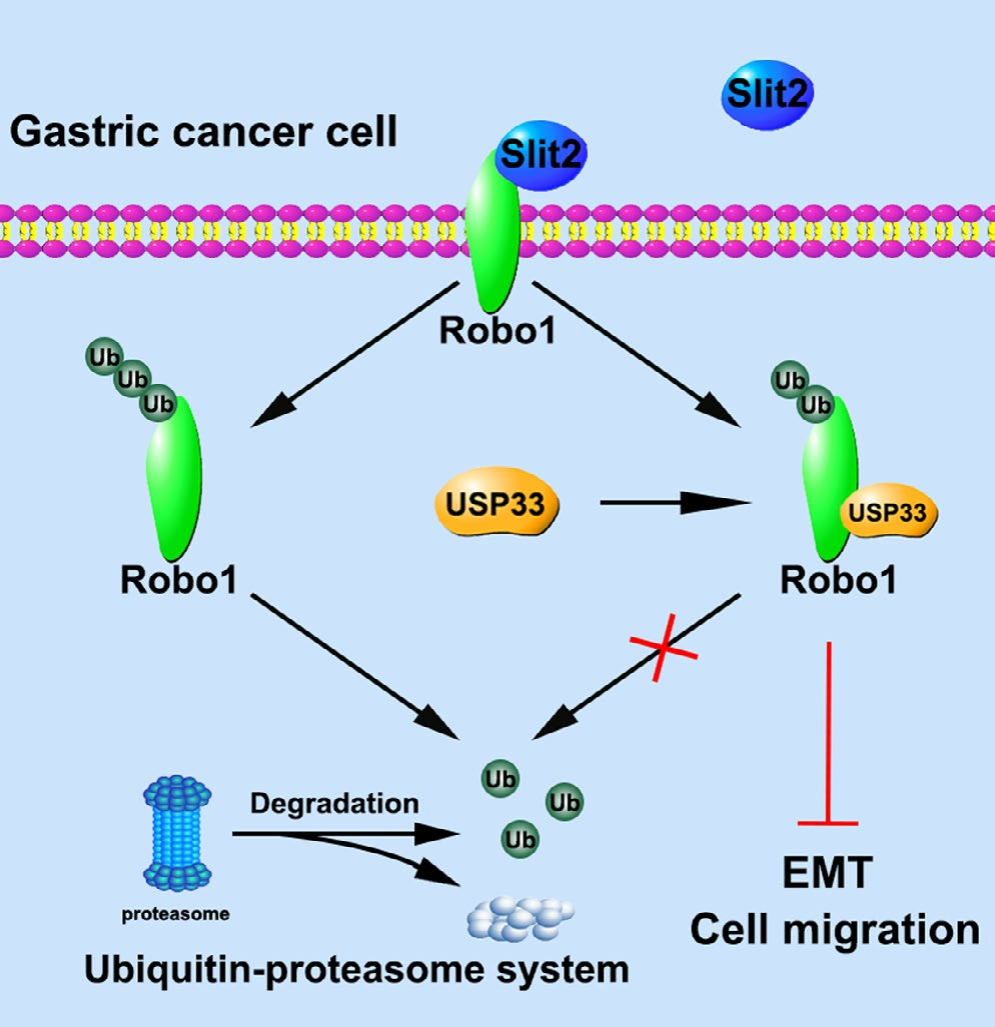
A diagram depicting a working model for USP33 in mediating the inhibitory function of Slit2‐Robo1 signalling in GC cells. In GC cells, Slit2 binds to Robo1 and inhibits cell migration and EMT. USP33 interacts with Robo1 and mediates Slit2‐Robo1 signalling by deubiquitinating Robo1 and preventing it from ubiquitin‐proteasome‐mediated degradation

In this study, we first report the involvement of Slit‐Robo signalling in the EMT of GC. We also demonstrate the mechanism of how USP33 mediates Slit‐Robo signalling in GC. As illustrated in Figure 7, USP33 interacts with Robo1 and stabilizes Robo1, preventing it from ubiquitin‐proteasome‐mediated degradation.

The first indication that Slit‐Robo signalling might play an important role in cancer derived from studies by Sundaresan and colleagues, which identified and cloned the DUTT1 gene (later renamed as ROBO1) and used probes to detect two homozygous deletions at the 3p12 locus in lung and breast carcinomas.^36^, ^37^ Subsequent studies have confirmed the involvement of Slit‐Robo signalling in several types of cancer.^14^, ^17^, ^38^, ^39^, ^40^ Overwhelming evidence suggests that Slit expression is reduced in different types of cancers.^38^, ^39^ However, the role of Slit‐Robo signalling in GC remains controversial. For example, it was reported that POU2F2 promotes GC metastasis through a positive regulation of Robo1,^19^ whereas another study showed that down‐regulating Slit2 increases growth and motility of GC cells by activating AKT/β‐catenin.^17^ In our study, we clearly demonstrate the downregulation of Slit2 and Robo1 expression in multiple datasets and our samples at both mRNA and protein levels. Our data indicate that Slit2 inhibits the migration of GC cell in a Robo‐dependent manner. This is consistent with our previous studies of lung cancer^16^ and breast cancer^15^ and with other studies such as medulloblastoma^43^ and glioma.^44^ Moreover, we found that Slit2 inhibits the EMT process, which may support for the clinical application of Slit‐Robo signalling.

Several Robo‐interacting molecules, such as srGAP,^8^ Abl,^9^ ERK1/2,^10^ USP33^15^, ^22^ and Myo9b,^16^ have been found to mediate Slit‐Robo signalling by different mechanisms. USP33 was initially identified as a substrate molecule which binds to VHL E3 ligase.^21^ To date, a considerable number of proteins interacting with USP33 have been identified, including beta‐arrestin,^45^ hSP56,^46^ RALB,^47^ ADRB^48^ and DIO2.^49^ The findings of these studies suggest that USP33 possesses biological functions critical for a wide range of human physiological and pathological processes. As a Robo1‐interacting protein, our previous studies have demonstrated that USP33 regulates the expression of Robo1^15^, ^27^, ^28^ and is required for Slit‐Robo signalling in modulating axon midline crossing^22^ and inhibiting cell migration in breast cancer,^15^ colorectal cancer^27^ and lung cancer.^28^ In this study, the results that Robo1 expression is reduced in GC prompted us to explore whether USP33 affect the Slit‐Robo signalling in GC. We also found that knock‐down of USP33 reduced the protein level of Robo1, while failed to affect Robo1 mRNA level, suggesting that USP33 regulates Robo1 protein expression through the post‐translational modification. Subsequent experiments proved the hypothesis that Robo1 is degraded mainly via the ubiquitin‐proteasome system. USP33 stabilizes Robo1 by reducing the ubiquitination of Robo1, thus is required for the Slit2‐Robo1 signalling in inhibiting gastric cancer cell migration and EMT. To our knowledge, it is the first study investigating the molecular mechanisms of USP33 in GC.

Transforming growth factor‐β (TGF‐β) has been proved as a critical factor during malignant progression in many types of cancer; meanwhile, the increased level and tumour‐promoting function of TGF‐β in gastric cancer have also been reported.^50^, ^51^ Furthermore, TGF‐β signalling is closely related to EMT and contributes to distant metastatic of tumours^32^, ^53^ and Smad protein phosphorylation is a key step during the activation TGF‐β signalling.^54^, ^55^ In this study, by the gene set enrichment analysis and Western blot of the key proteins, we demonstrated that the inhibitory functions of Slit2‐Robo1 on cell migration and EMT are mediated partially by the inactivation of TGF‐β signalling and USP33 is required for these effects.

The degradation of many intracellular short‐lived proteins relies on the ubiquitin‐proteasome system (UPS).^56^ The therapy targeting the ubiquitin system has developed into a promising strategy for cancer treatment.^57^ Data from our patient samples together with analyses of multiple independent datasets show that higher USP33 expression is significantly associated with longer patient survival, suggesting the potential applications of USP33 for GC therapy and predicting prognosis. Future studies are needed to investigate the potential value of Slit2‐Robo1‐USP33 in diagnosis and treatment of GC.

In summary, our data reveal the new molecular mechanism of USP33 in GC and Slit2‐Robo1‐USP33 pathway in suppressing GC cell migration and EMT. In addition, higher USP33 expression is significantly associated with extended patient survival. These results support the suppressive role of USP33 in GC and suggest the potential of USP33 as a prognostic biomarker and therapeutic target for GC.

## CONFLICT OF INTEREST

The authors have no conflicts of interest to declare.

## AUTHOR CONTRIBUTIONS

Xu ZK and Wu JY designed the study; Xia YW, Wang LJ and Xu ZP performed the cellular and animal experiments; Kong RR and Wang F performed bioinformatic analysis; Yin K, He ZY and Wang L collected the clinical samples; Xu H, Zhang DC and Yang L analysed the data; Xia YW, Wang LJ and Li BW wrote manuscript. All authors read and approved the final manuscript.
