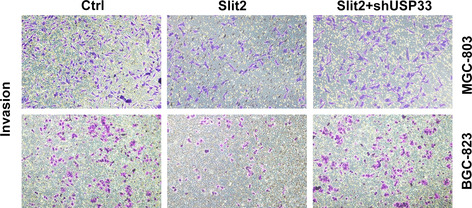# Correction to “Reduced USP33 expression in gastric cancer decreases inhibitory effects of Slit2‐Robo1 signalling on cell migration and EMT”

**DOI:** 10.1111/cpr.13632

**Published:** 2024-03-15

Xia Y , Wang L , Xu Z , Kong R , Wang F , Yin K , Xu J , Li B , He Z , Wang L , Xu H , Zhang D , Yang L , Wu JY , Xu Z . Reduced USP33 expression in gastric cancer decreases inhibitory effects of Slit2‐Robo1 signalling on cell migration and EMT. Cell Prolif 2019 May;52(3):e12606.30896071 10.1111/cpr.12606PMC6536419

The images of MGC803 Transwell assays in Figure 5F are incorrect. The three images of MGC803, which were inadvertently placed in Figure 5F, belong to three visual fields of the same Transwell chamber. Corrected images for Figure 5F are provided below. The corrections have no impact on the results of the study. We apologize for this error.