# Supplementary figures and images for: Reduced USP33 expression in gastric cancer decreases inhibitory effects of Slit2‐Robo1 signalling on cell migration and EMT
Source: Cell Prolif. 2019 Mar 21;52(3):e12606. doi: 10.1111/cpr.12606 (PMC6536419; doi:10.1111/cpr.12606)

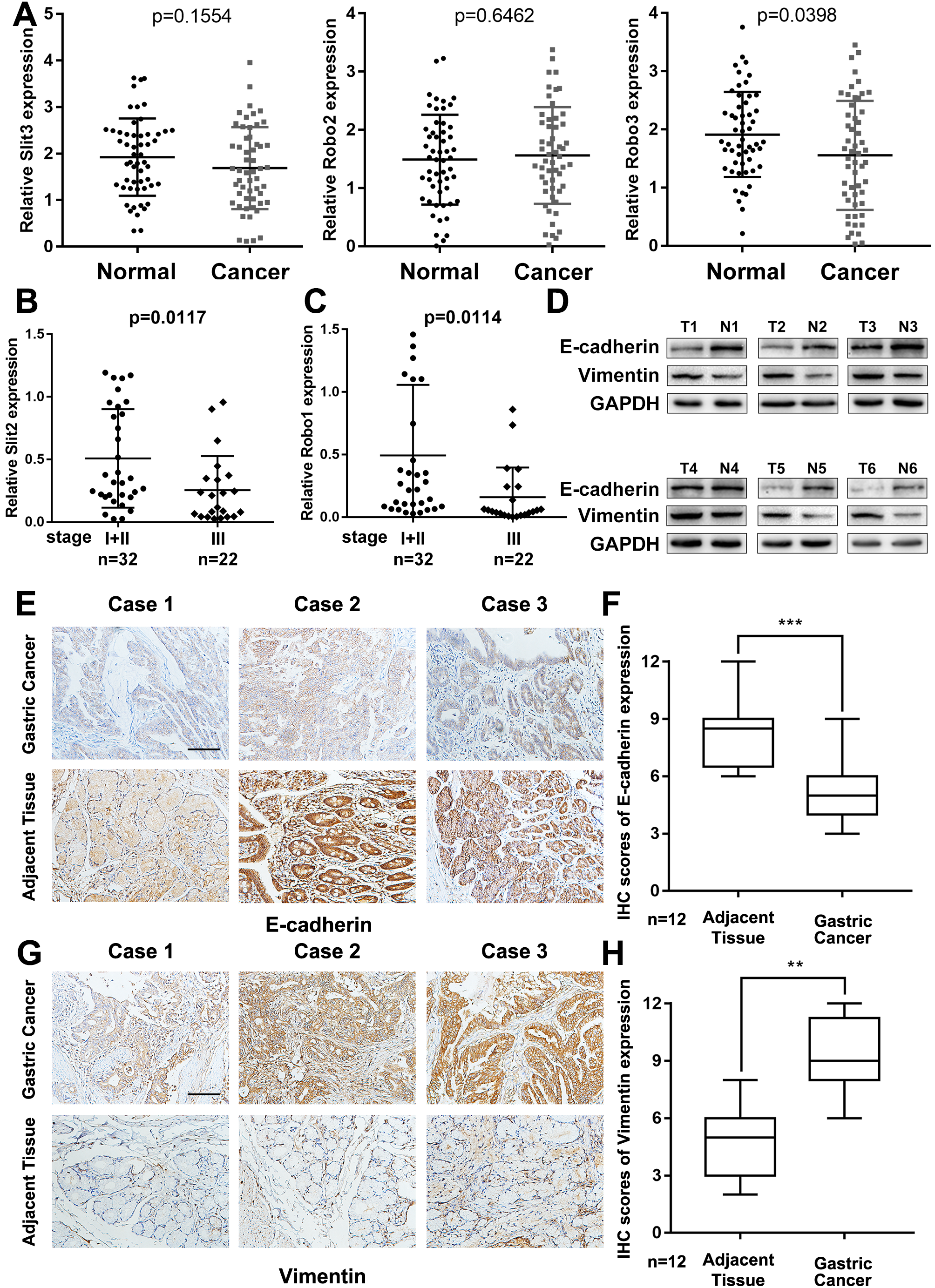

Supplement: Supplementary file 1 [file CPR-52-e12606-s001.tif]

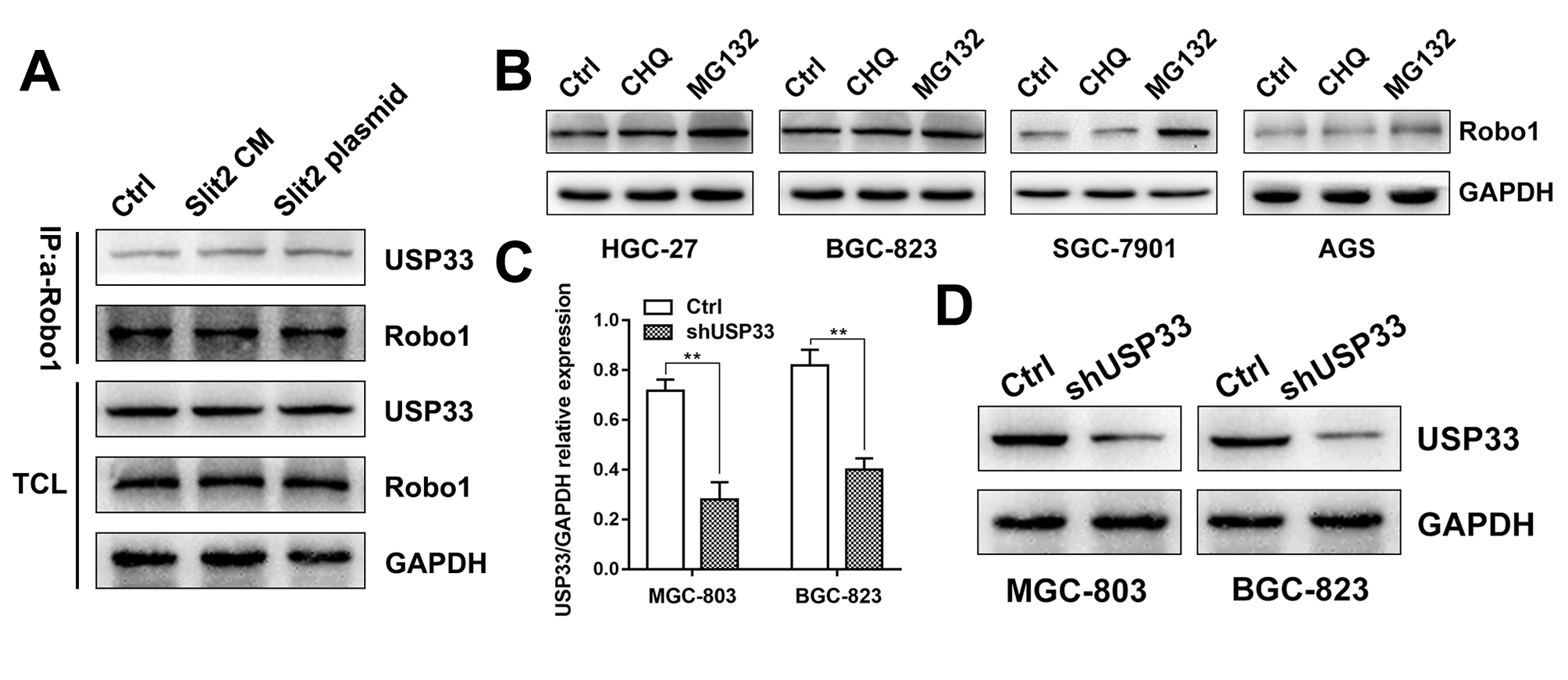

Supplement: Supplementary file 2 [file CPR-52-e12606-s002.tif]
