# Supplementary material for: Reduced USP33 expression in gastric cancer decreases inhibitory effects of Slit2‐Robo1 signalling on cell migration and EMT
Source: Cell Prolif. 2019 Mar 21;52(3):e12606. doi: 10.1111/cpr.12606 (PMC6536419; doi:10.1111/cpr.12606)
Supplement: Supplementary file 3 [file CPR-52-e12606-s003.docx]

**Supplementary figure legends**

**Figure S1 E-Cadherin was decreased while Vimentin was elevated in GC samples.**

**(A)** Relative mRNA expression of Slit3, Robo2 and Robo3 in 54 paired GC and adjacent tissues analyzed by qRT-PCR. **(B)** Relative mRNA expression of Slit2 in stage I+II group and stage III group. **(C)** Relative mRNA expression of Robo1 in stage I+II group and stage III group. **(D)**E-cadherin and Vimentin protein levels in 6 random paired GC and adjacent tissues were determined by Western blotting. GAPDH was used as the internal control. **(E)** Representative images of immunohistochemical (IHC) staining of E-cadherin in 12 paired GC and adjacent tissues. Original magnification, 200×; scale bar = 100 µm. **(F)** Box plots showing the IHC scores for E-cadherin protein expression, analyzed by Mann-Whitney U-test. **(G)** Representative images of immunohistochemical (IHC) staining of Vimentin in 12 paired GC and adjacent tissues. Original magnification, 200×; scale bar = 100 µm. **(H)** Box plots showing the IHC scores for Vimentin protein expression, analyzed by Mann-Whitney U-test.

**Figure S2 Assessing the pattern of Robo1 degradation in GC cell lines.**

**(A)** Coimmunoprecipitation was carried out in untreated MGC-803 cells, cells treated with Slit2 containing media, or cells transfected with Slit2 plasmid. Robo1 and USP33 were detected in immunoprecipitated proteins by Western blotting. CM: containing media. **(B)** HGC-27, BGC-823, SGC-7901 or AGS were left untreated, treated with Chloroquine (CHQ, 50 μM, 10h) or MG132 (10 μM, 10h), Robo1 was detected by Western blotting. GAPDH was used as the internal control. **(C)** Relative USP33 mRNA levels in MGC-803 and BGC-823 transfected with shUSP33 or shControl were examined by qRT-PCR. **(D)** Protein levels of USP33 in MGC-803 and BGC-823 transfected with shUSP33 or shControl were detected by Western blotting. **(E)** The migration of MGC-803 and BGC-823 examined by wound healing assays transfected with shControl or shUSP33. Original magnification, 40×; scale bar = 100 µm. **(F)** Quantification of the distance of MGC-803 and BGC-823 cell migration. **(G)** The migratory capabilities of MGC-803 and BGC-823 transfected with shControl or shUSP33 were determined by Transwell assays. Original magnification, 100×; scale bar = 200 µm. **(H)** Cell migration was quantified. **(I)** The invasive capabilities of MGC-803 and BGC-823 tested by Transwell assays. **(J)** Cell invasion was quantified.
